# Jangwon-hwan Improves Memory Performance in Mice with Enhanced Cholinergic Signaling and Synaptic Plasticity

**DOI:** 10.3390/ph19091479

**Published:** 2026-09-17

**Authors:** Seungmin Lee, Minji Lee, Jin Hee Kim, Yujin Choi, Kilyong Bae, Myung Sook Oh

**Affiliations:** 1 Department of Biomedical and Pharmaceutical Sciences, Graduate School, Kyung Hee University, 26 Kyungheedae-ro, Dongdaemun-gu, Seoul 02447, Republic of Korea; smlee0817@khu.ac.kr (S.L.); alswl3736@khu.ac.kr (M.L.); jinhee1231@khu.ac.kr (J.H.K.); yj001217@khu.ac.kr (Y.C.); 2 Pharmaceutical Development Department, Kwangdong Pharmaceutical Co., Ltd., Gwacheon-si 13840, Gyeonggi-do, Republic of Korea; kilyong.bae@ekdp.com; 3 Department of Traditional Korean Pharmacy and Integrative Pharmaceutical Sciences, College of Pharmacy, Kyung Hee University, 26 Kyungheedae-ro, Dongdaemun-gu, Seoul 02447, Republic of Korea

**Keywords:** Jangwon-hwan, traditional medicine, memory enhancing, synaptic plasticity, cholinergic system

## Abstract

**Background/Objectives**: Jangwon-hwan (JH), known as Zhuangyuan-wan in Chinese medicine, has traditionally been used for forgetfulness and poor memory, often accompanied by palpitations and insomnia. However, the pharmacological mechanisms underlying its traditional use for memory-related symptoms remain unclear. This study aimed to evaluate the effects of JH on memory function and to elucidate the underlying mechanisms, thereby providing scientific evidence for its use. **Methods**: JH (30 or 100 mg/kg) was orally administered for 28 days in normal mice and for 14 days in scopolamine-induced memory-impaired mice. Memory performance was assessed behaviorally, with molecular and histological analyses of brain tissue. **Results**: JH improved passive avoidance memory and increased hippocampal synaptophysin and postsynaptic density protein 95 in normal mice. JH also enhanced medial septal cholinergic neuronal activation and increased hippocampal acetylcholine levels. It further increased muscarinic acetylcholine receptor M1 and metabotropic glutamate receptor 5 expression, upregulated synaptic plasticity-related genes, and enhanced hippocampal neuronal activation. Consistent with its effects on cholinergic signaling, JH attenuated scopolamine-induced memory impairment and preserved hippocampal phosphorylation of calcium/calmodulin-dependent protein kinase II, extracellular signal-regulated kinase, and cAMP response element-binding protein. **Conclusions**: Collectively, these findings indicate that the memory-enhancing effects of JH are associated with enhanced cholinergic signaling, synaptic plasticity, and hippocampal neuronal activation, providing molecular evidence supporting its traditional use for forgetfulness and poor memory.

## 1. Introduction

Memory is the process by which information acquired from external stimuli is encoded, stored within the nervous system, and retrieved when necessary [1,2]. The hippocampus plays a pivotal role in receiving newly acquired information and converting short-term memory into long-term memory through memory consolidation, thereby enabling stable storage within neural circuits [3,4]. Intact memory function is essential for adaptive behavior, learning, and the maintenance of personal identity and social independence [5]. However, memory function may progressively decline with aging, chronic stress, and diverse environmental factors [6,7]. Such a decline substantially disrupts daily functioning and reduces overall quality of life [8]. As the aging population increases, the prevalence of memory impairment rises, and in severe cases, it may progress to dementia [9,10]. Consequently, preserving and enhancing memory functions has become increasingly important.

Synaptic plasticity is central to the encoding and storage of memory and is essential for maintaining overall memory function [6,11]. This process refers to activity-dependent changes in synaptic efficacy and connectivity strength [12], which strengthen synaptic function and contribute to memory enhancement [13]. At the molecular level, synaptic plasticity involves activity-regulated gene expression and modulation of synaptic proteins, facilitating structural remodeling and stabilization of synaptic connections [14,15]. Notably, cholinergic neurotransmission plays a key regulatory role in coordinating these plastic changes [16]. This modulatory effect is primarily mediated by acetylcholine (ACh) [17]. In the hippocampus, ACh regulates neuronal responsiveness during learning and memory, and its receptor-mediated signaling is closely associated with intracellular pathways that govern synaptic plasticity [17,18,19]. Hippocampal cholinergic modulation is mainly supported by cholinergic projections from the medial septum (MS), forming the medial septum–hippocampal circuit [20]. This circuit contributes to learning and memory by regulating hippocampal activity and synaptic plasticity [21]. Within the hippocampus, the dentate gyrus plays an important role in memory encoding and discrimination [22]. Thus, enhancing cholinergic signaling and synaptic plasticity is critical for improving memory.

Jangwon-hwan (JH; Zhuangyuan-wan in Chinese), a traditional prescription recorded in *Donguibogam*, has been used to treat memory decline, forgetfulness, palpitation, and insomnia [23,24]. Previous pharmacological studies have demonstrated the neuroprotective effects of modified JH preparations. Specifically, LMK02, a modified seven-herb preparation derived from JH, attenuated amyloid-β (Aβ)_1–42_-induced neurotoxicity in SH-SY5Y neuroblastoma cells and reduced cerebral Aβ_1–40_ and Aβ_1–42_ levels and amyloid plaque deposition in Tg-APPswe/PS1dE9 mice [24]. LMK03, another modified preparation derived from JH, attenuated H_2_O_2_- and oligomeric Aβ_1–42_-induced cytotoxicity in SH-SY5Y cells and partially reduced cerebral Aβ_1–40_ and Aβ_1–42_ levels and amyloid plaque deposition in the same mouse model [25]. These modified JH preparations, including LMK02 and LMK03, were also summarized in a review of traditional herbal formulations for cognitive impairment [26]. Several constituent herbs of JH, including Ginseng Radix, Polygalae Radix, and Ziziphi Semen, have also been reported to exert memory-enhancing effects [27,28,29]. Additionally, Acori Graminei Rhizoma and Longan Arillus have been reported to ameliorate Aβ-induced cognitive deficits in mice [30,31]. Although previous studies have demonstrated the neuroprotective effects of modified JH preparations and several of its constituent herbs, the memory-enhancing effects of JH itself remain insufficiently explored. Moreover, previous studies have largely focused on Aβ-related neurotoxicity and amyloid pathology, and the pharmacological mechanisms underlying the effects of JH on memory function remain unclear.

This study aimed to investigate the memory-enhancing effects of JH and to explore its underlying mechanisms. Its effects were first evaluated in a scopolamine-induced memory impairment model. Subsequently, JH was administered to normal mice, and memory function was assessed using behavioral tests. To examine whether JH modulates cholinergic synaptic plasticity, analyses were conducted on synaptic integrity, cholinergic function, postsynaptic receptor regulation, plasticity-related gene expression, and neuronal activation in the mouse brain.

## 2. Results

### 2.1. Chromatographic Analysis and Quantification of Major Compounds in JH

**Figure 1:**
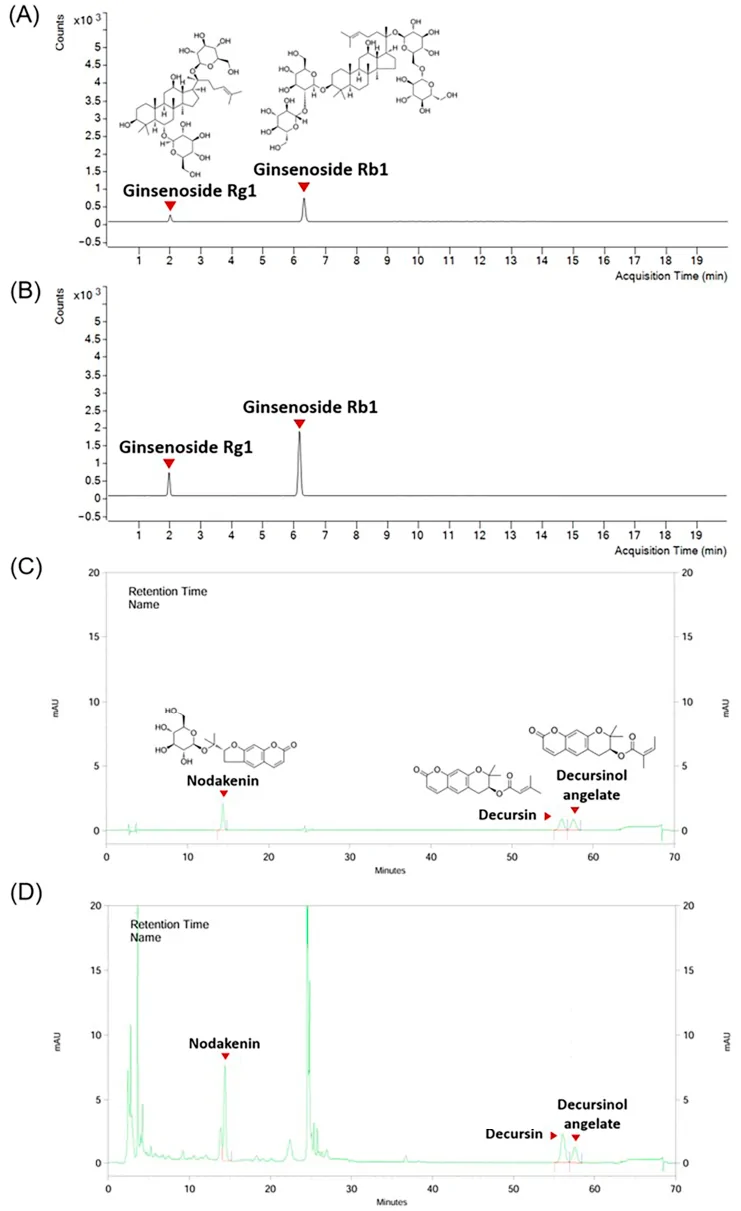
LC chromatograms of ginsenoside Rg1 (RT = 1.961 min) and ginsenoside Rb1 (RT = 6.169 min) standard solutions (**A**). LC–MS/MS chromatogram of JH (**B**). HPLC chromatograms of nodakenin (RT = 14.377 min), decursin (RT = 56.107 min), and decursinol angelate (RT = 57.587 min) standard solutions (**C**). HPLC-UV chromatogram of JH (**D**) at 330 nm. Abbreviations: LC, liquid chromatography; RT, retention time; MS/MS, tandem mass spectrometry; JH, Jangwon-hwan; HPLC, high-performance liquid chromatography; UV, ultraviolet.

Chromatographic analyses were conducted to characterize and quantify major compounds in JH. Liquid chromatography-tandem mass spectrometry (LC–MS/MS) analysis identified and quantified ginsenoside Rg1 and ginsenoside Rb1, with contents in JH of 91 and 83 μg/g, respectively (Figure 1A,B). The corresponding multiple reaction monitoring (MRM) transition chromatograms and product ion spectra are provided in Supplementary Figure S1. High-performance liquid chromatography (HPLC)-ultraviolet (UV) analysis further identified and quantified nodakenin, decursin, and decursinol angelate, with contents in JH of 804, 397, and 221 μg/g, respectively (Figure 1C,D). Additional qualitative LC–MS/MS analysis confirmed the presence of Liriope muscari saponin C (Supplementary Figure S2), spinosin, harpagoside, and tenuifolin in JH by comparison with authentic reference standards (Supplementary Figure S3).

### 2.2. JH Improved Memory Performance and Upregulated Hippocampal Synaptic Protein Expression and ACh Levels

**Figure 2:**
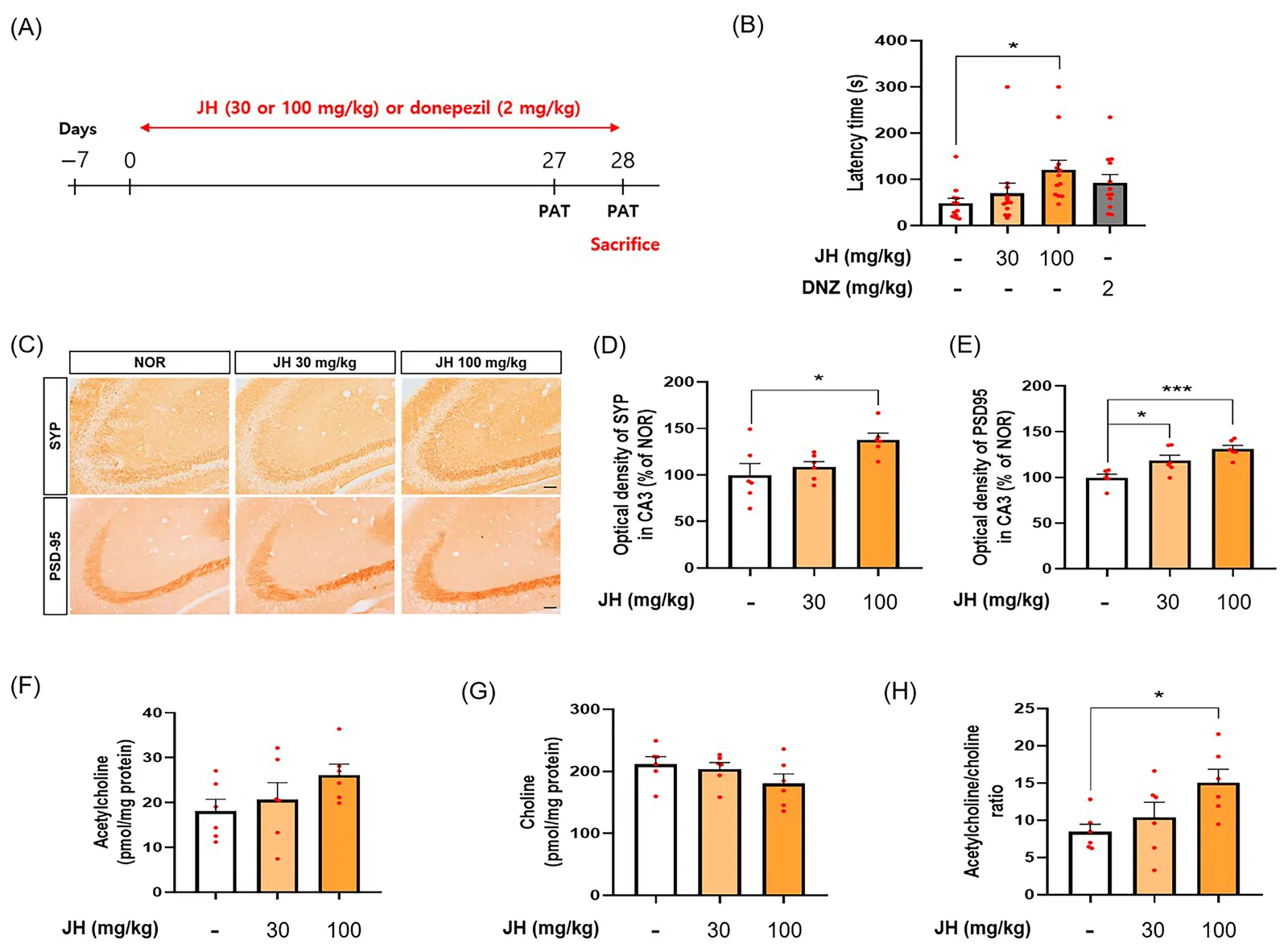
Memory-enhancing effects of JH in normal mice. Timeline of drug administration and behavioral testing (**A**). JH effects on memory function were assessed using the PAT (**B**). Representative immunostaining images (**C**) and quantitative analysis of SYP (**D**) and PSD-95 (**E**) in the CA3 region (*n* = 6 per group). Hippocampal ACh and choline levels and the ACh/choline ratio (**F**–**H**). Data were analyzed using ANOVA with Dunnett’s post hoc test. *** *p* < 0.001 and * *p* < 0.05 vs. normal group. Scale bar  =  100 μm. Abbreviations: JH, Jangwon-hwan; DNZ, donepezil; PAT, passive avoidance test; SYP, synaptophysin; PSD-95, postsynaptic density protein 95; CA3, cornu ammonis 3; ACh, acetylcholine; ANOVA, analysis of variance.

To evaluate the memory-enhancing effects of JH under normal physiological conditions, JH (30 or 100 mg/kg) was administered to normal mice for 28 days. Donepezil (DNZ) served as a positive control [32]. In the passive avoidance test (PAT), one-way analysis of variance (ANOVA) showed a trend toward a group difference in step-through latency [F(3, 44) = 2.738; *p* = 0.0547]. Dunnett’s post hoc test showed that JH at 100 mg/kg significantly increased step-through latency compared with the normal group (*p* = 0.0244; Figure 2B). DNZ treatment also resulted in a numerical increase in step-through latency compared with the normal group (Figure 2B).

For hippocampal synaptic markers, one-way ANOVA revealed significant group differences in synaptophysin (SYP) expression [F(2, 15) = 5.003; *p* = 0.0216] and postsynaptic density protein 95 (PSD95) expression [F(2, 15) = 11.58; *p* = 0.0009], followed by Dunnett’s post hoc test. JH at 100 mg/kg significantly increased SYP expression in the cornu ammonis 3 (CA3) region of the hippocampus compared with the normal group (*p* = 0.0161; Figure 2D). In addition, JH significantly increased PSD95 expression at both 30 mg/kg (*p* = 0.0220) and 100 mg/kg (*p* = 0.0005) compared with the normal group (Figure 2E).

Hippocampal ACh and choline levels were measured using an enzyme-linked immunosorbent assay (ELISA) kit. One-way ANOVA revealed no significant group differences in ACh [F(2, 15) = 1.858; *p* = 0.1901] or choline levels [F(2, 15) = 1.593; *p* = 0.2359] (Figure 2F,G). In contrast, a significant group difference was observed in the ACh/choline ratio [F(2, 15) = 4.078; *p* = 0.0385]. Dunnett’s post hoc test showed that JH at 100 mg/kg significantly increased the ACh/choline ratio compared with the normal group (*p* = 0.0259; Figure 2H).

### 2.3. JH Increased ChAT-Positive Neurons and Enhanced Cholinergic Neuronal Activation in the MS

**Figure 3:**
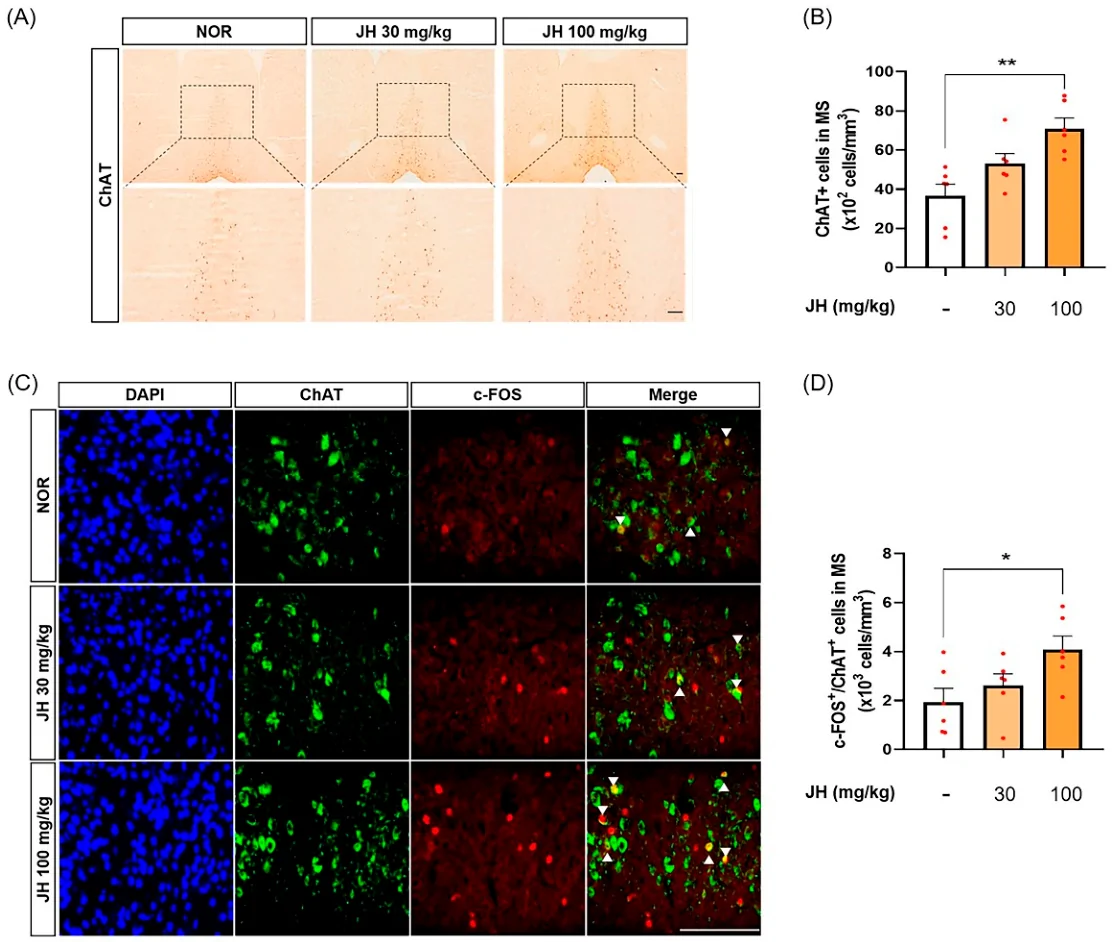
Effects of JH on ChAT-positive neurons and cholinergic activation in the MS. Representative images (**A**) and quantitative analysis of ChAT-positive cells (**B**) are shown (*n* = 6 per group). Immunofluorescence images of ChAT (green), c-FOS (red), and DAPI (blue) in the MS (**C**), and quantitative analysis of ChAT/c-FOS double-positive cells (**D**) performed using ImageJ (version 1.38x) are shown (*n* = 6 per group). Data were analyzed using one-way ANOVA with Dunnett’s post hoc test. ** *p* < 0.01 and * *p* < 0.05 vs. normal group. Scale bar  =  100 μm. Abbreviations: JH, Jangwon-hwan; ChAT, choline acetyltransferase; MS, medial septum; c-FOS, cellular Fos proto-oncogene; DAPI, 4′,6-diamidino-2-phenylindole; ANOVA, analysis of variance.

To investigate whether JH modulates cholinergic neuronal activity, choline acetyltransferase (ChAT)-positive neurons in the MS were quantified. One-way ANOVA revealed a significant group difference in the number of ChAT-positive cells in the MS [F(2, 15) = 9.548; *p* = 0.0021], followed by Dunnett’s post hoc test. JH at 100 mg/kg significantly increased the number of ChAT-positive cells compared with the normal group (*p* = 0.0011; Figure 3B).

Double immunofluorescence staining for ChAT and cellular Fos proto-oncogene (c-FOS) was performed to assess cholinergic neuronal activation in the MS. One-way ANOVA revealed a significant group difference in the number of ChAT/c-FOS double-positive cells [F(2, 15) = 4.281; *p* = 0.0338], followed by Dunnett’s post hoc test. JH at 100 mg/kg significantly increased the number of ChAT/c-FOS double-positive cells in the MS compared with the normal group (*p* = 0.0220; Figure 3D).

### 2.4. JH Upregulated Hippocampal CHRM1, mGluR5, and Synaptic Plasticity-Related Markers

**Figure 4:**
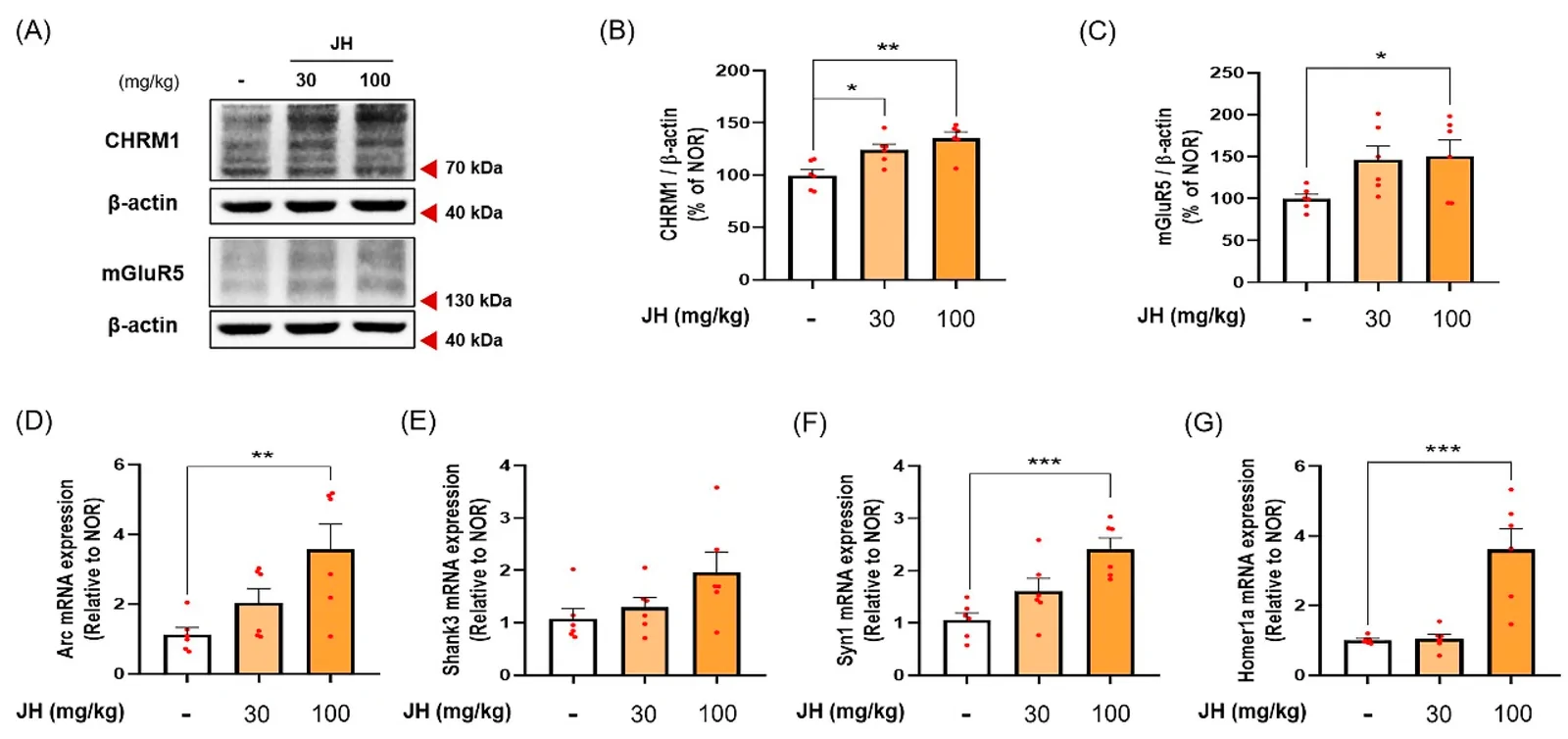
Effects of JH on synaptic plasticity-related markers in the hippocampus. Representative Western blot images (**A**) and quantitative analyses of CHRM1 and mGluR5 normalized to β-actin (**B**,**C**) are shown (*n* = 6 per group). Quantitative analyses of Arc, Shank3, Syn1, and Homer1a mRNA levels, with GAPDH used as an internal control (**D**–**G**), are presented (*n* = 6 per group). Data were analyzed using one-way ANOVA with Dunnett’s post hoc test or unpaired Student’s *t*-test. *** *p* < 0.001, ** *p* < 0.01, and * *p* < 0.05 vs. normal group. Abbreviations: JH, Jangwon-hwan; CHRM1, cholinergic receptor muscarinic 1; mGluR5, metabotropic glutamate receptor 5; Arc, activity-regulated cytoskeleton-associated protein; Shank3, SH3 and multiple ankyrin repeat domains 3; Syn1, synapsin I; Homer1a, Homer scaffold protein 1a; GAPDH, glyceraldehyde-3-phosphate dehydrogenase; ANOVA, analysis of variance.

Cholinergic receptor muscarinic 1 (CHRM1) and metabotropic glutamate receptor 5 (mGluR5) are receptors involved in cholinergic and glutamatergic signaling, both of which are associated with synaptic plasticity [33,34]. Additionally, Activity-regulated cytoskeleton-associated protein (Arc), SH3 and multiple ankyrin repeat domains 3 (Shank3), synapsin 1 (Syn1), and homer scaffold protein 1a (Homer1a) are synaptic plasticity-related factors associated with synaptic structure and activity-dependent neuronal signaling [35]. To investigate the molecular mechanisms underlying JH-induced memory enhancement, the expression levels of these proteins and genes were analyzed in the hippocampus. One-way ANOVA revealed a significant group difference in CHRM1 protein levels [F(2, 15) = 10.01; *p* = 0.0017], followed by Dunnett’s post hoc test. JH significantly increased CHRM1 expression at both 30 mg/kg (*p* = 0.0162) and 100 mg/kg (*p* = 0.0011) compared with the normal group (Figure 4B). For mGluR5 protein levels, one-way ANOVA showed a trend toward a group difference [F(2, 15) = 3.659; *p* = 0.0508]. Dunnett’s post hoc test showed that JH at 100 mg/kg significantly increased mGluR5 expression compared with the normal group (*p* = 0.0493; Figure 4C).

For synaptic plasticity-related genes, one-way ANOVA revealed significant group differences in Arc [F(2, 15) = 6.282; *p* = 0.0104], Syn1 [F(2, 15) = 11.01; *p* = 0.0011], and Homer1a [F(2, 15) = 17.41; *p* = 0.0001], but not in Shank3 [F(2, 15) = 2.866; *p* = 0.0883]. Dunnett’s post hoc test showed that JH significantly increased the hippocampal mRNA expression levels of Arc (*p* = 0.0060; Figure 4D), Syn1 (*p* = 0.0006; Figure 4F), and Homer1a (*p* = 0.0002; Figure 4G) compared with the normal group. Shank3 expression showed a tendency toward an increase following JH treatment (Figure 4E).

### 2.5. JH Increased Neuronal Activation and c-FOS Expression in the Hippocampus

**Figure 5:**
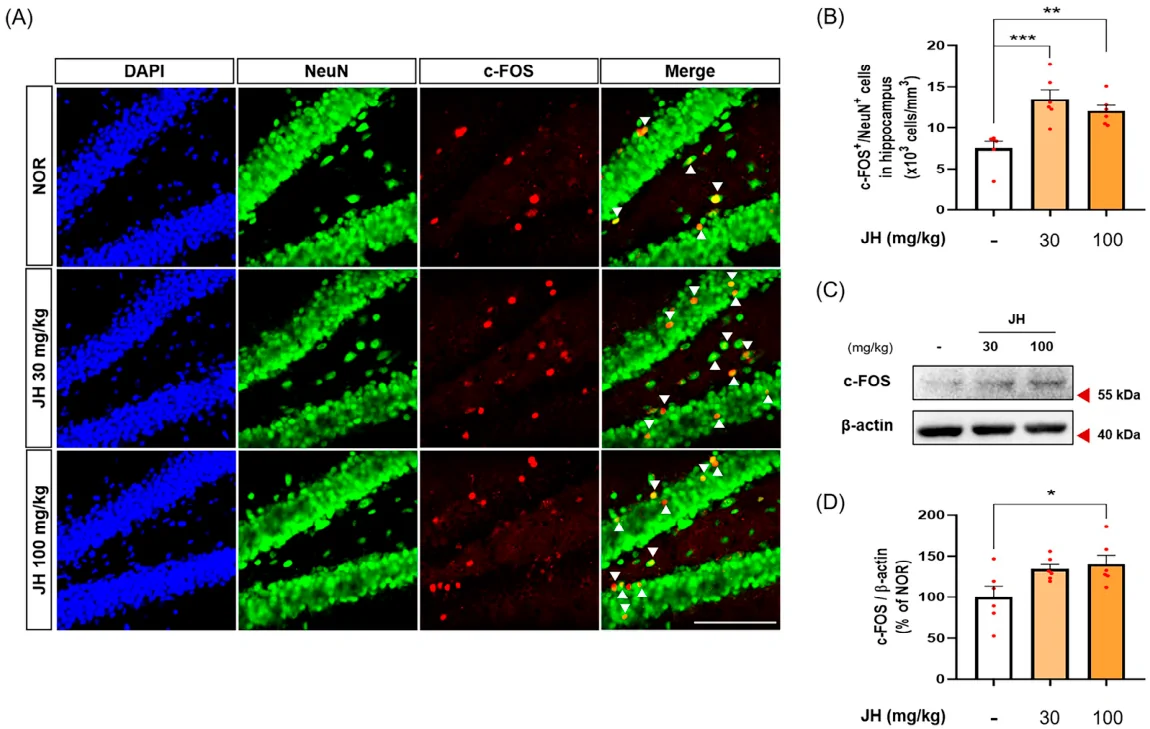
Effects of JH on hippocampal neuronal activation and c-FOS expression. Immunofluorescence images of NeuN (green), c-FOS (red), and DAPI (blue) in the DG (**A**) and quantitative analysis of NeuN/c-FOS double-positive cells (**B**) performed using ImageJ (*n* = 6 per group). Representative Western blot images (**C**) and quantitative analysis of c-FOS normalized to β-actin (**D**) are shown (*n* = 6 per group). Data were analyzed using one-way ANOVA with Dunnett’s post hoc test. *** *p* < 0.001, ** *p* < 0.01, and * *p* < 0.05 vs. normal group. Scale bar = 100 μm. Abbreviations: JH, Jangwon-hwan; NeuN, neuronal nuclei; c-FOS, Fos proto-oncogene; DAPI, 4′,6-diamidino-2-phenylindole; DG, dentate gyrus; ANOVA, analysis of variance.

To determine whether JH-induced enhancement of synaptic plasticity is accompanied by increased neuronal activity, c-FOS expression was evaluated in the dentate gyrus (DG) of the hippocampus. One-way ANOVA revealed a significant group difference in the number of neuronal nuclei (NeuN)/c-FOS double-positive cells [F(2, 15) = 12.10; *p* = 0.0007], followed by Dunnett’s post hoc test. JH significantly increased the number of NeuN/c-FOS double-positive cells at both 30 mg/kg (*p* = 0.0006) and 100 mg/kg (*p* = 0.0052) compared with the normal group (Figure 5B).

Hippocampal c-FOS protein levels were also analyzed by Western blotting. One-way ANOVA revealed a significant group difference in c-FOS protein levels [F(2, 15) = 4.270; *p* = 0.0340], followed by Dunnett’s post hoc test. JH at 100 mg/kg significantly increased hippocampal c-FOS protein levels compared with the normal group (*p* = 0.0295; Figure 5D).

### 2.6. JH Prevented Scopolamine-Induced Memory Deficits and Upregulated Phosphorylation of Hippocampal CaMKII/ERK/CREB

**Figure 6:**
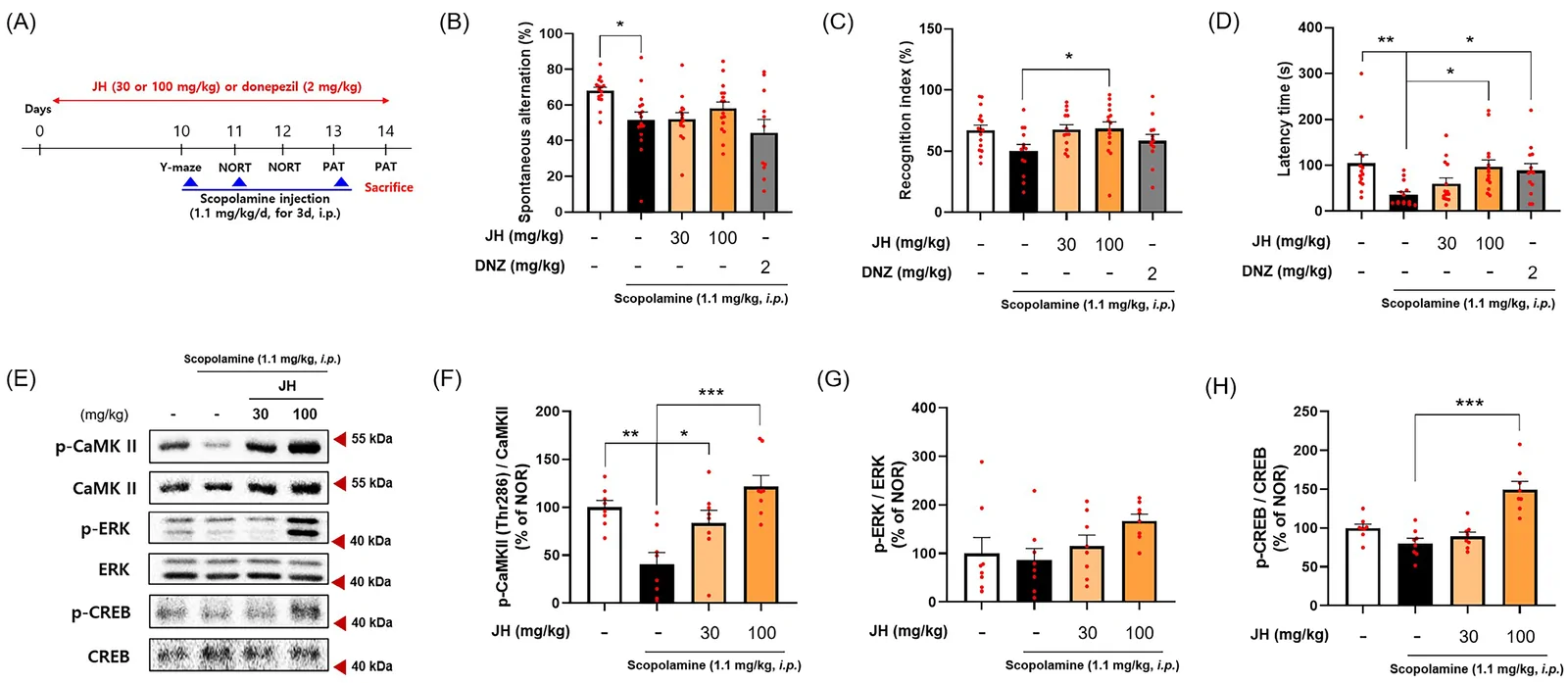
Experimental scheme and the effects of JH on scopolamine-induced memory impairment. Timeline of drug administration and behavioral testing (**A**). JH effects on memory function were assessed using the Y-maze (**B**), NORT (**C**), and PAT (**D**) (*n* = 13–16 per group). Representative Western blot images (**E**) and quantitative analyses of phosphorylated CaMKII, ERK, and CREB normalized to total protein levels (**F**–**H**) are shown (*n* = 8 per group). Data were analyzed using one-way ANOVA with Dunnett’s post hoc test. *** *p* < 0.001, ** *p* < 0.01, and * *p* < 0.05 vs. the scopolamine-only group. Abbreviations: JH, Jangwon-hwan; DNZ, donepezil; NORT, novel object recognition test; PAT, passive avoidance test; CaMKII, calcium/calmodulin-dependent protein kinase II; ERK, extracellular signal-regulated kinase; CREB, cAMP response element-binding protein; ANOVA, analysis of variance.

To investigate whether JH prevents memory deficits, JH (30 or 100 mg/kg) was administered to scopolamine-injected mice. DNZ, an acetylcholinesterase inhibitor, served as a positive control [36]. Memory performance was evaluated using the Y-maze, novel object recognition test (NORT), and PAT. One-way ANOVA revealed significant group differences in spontaneous alternation in the Y-maze test [F(4, 68) = 4.380; *p* = 0.0033], recognition index in the NORT [F(4, 65) = 2.557; *p* = 0.0469], and step-through latency in the PAT [F(4, 64) = 4.013; *p* = 0.0058]. Dunnett’s post hoc test showed that scopolamine-treated mice exhibited significantly reduced spontaneous alternation in the Y-maze test (*p* = 0.0159; Figure 6B) and step-through latency in the PAT (*p* = 0.0042; Figure 6D) compared with the normal group, while the recognition index in the NORT was also reduced. Compared with the scopolamine group, JH at 100 mg/kg significantly increased the recognition index in the NORT (*p* = 0.0338; Figure 6C) and step-through latency in the PAT (*p* = 0.0122; Figure 6D). DNZ also significantly increased step-through latency compared with the scopolamine group (*p* = 0.0427; Figure 6D).

To determine whether JH upregulates the calcium/calmodulin-dependent protein kinase II (CaMKII)/extracellular signal-regulated kinase (ERK)/cAMP response element-binding protein (CREB) signaling cascade, which is critical for synaptic plasticity and memory consolidation [37], hippocampal phosphorylation levels of CaMKII, ERK, and CREB were measured. One-way ANOVA revealed significant group differences in p-CaMKII/CaMKII [F(3, 28) = 9.288; *p* = 0.0002] and p-CREB/CREB levels [F(3, 28) = 18.49; *p* < 0.0001], followed by Dunnett’s post hoc test. Scopolamine significantly reduced CaMKII phosphorylation compared with the normal group (*p* = 0.0024; Figure 6F). Compared with the scopolamine group, JH significantly increased CaMKII phosphorylation at both 30 mg/kg (*p* = 0.0300) and 100 mg/kg (*p* < 0.0001; Figure 6F). JH at 100 mg/kg also significantly increased CREB phosphorylation compared with the scopolamine group (*p* < 0.0001; Figure 6H). In contrast, no significant group difference was observed in p-ERK/ERK levels [F(3, 28) = 2.094; *p* = 0.1236], although JH at 100 mg/kg showed a tendency toward increased ERK phosphorylation (Figure 6G).

## 3. Discussion

The present study provides evidence that JH improves memory-related behavioral performance under both scopolamine-induced cholinergic dysfunction and normal physiological conditions. These effects appear to involve coordinated regulation of hippocampal memory-related signaling, septo-hippocampal cholinergic modulation, synaptic plasticity-related markers, and neuronal activation. Thus, the memory-improving effect of JH may be associated with the combined modulation of cholinergic and synaptic plasticity-related mechanisms.

The septo-hippocampal cholinergic system is a key regulatory circuit underlying hippocampal-dependent memory processing [38]. This circuit modulates hippocampal neuronal activity and synaptic responsiveness during memory formation [39]. Cholinergic neurons in the MS provide ACh to the hippocampus, influencing information processing [40]. These neurons express ChAT, the biosynthetic enzyme responsible for ACh production and a phenotypic marker of cholinergic neurons [41]. JH increased the number of ChAT-positive neurons in the MS, indicating an enhancement of the cholinergic neuronal population. JH also increased ChAT and c-FOS co-localization in the MS, suggesting functional activation of septal cholinergic neurons. Consistent with this upstream activation, JH significantly elevated hippocampal ACh levels. ACh plays a critical role in memory formation by regulating hippocampal excitability and synaptic signaling. These findings suggest that JH may enhance septo-hippocampal cholinergic tone by increasing the activation of medial septal cholinergic neurons, thereby supporting hippocampal conditions favorable for memory processing.

Elevated ACh interacts with muscarinic receptors in the hippocampus, particularly CHRM1, the predominant subtype mediating cholinergic modulation of hippocampal neuronal activity and synaptic function [42]. JH upregulated CHRM1 expression in the hippocampus, which, in the context of elevated ACh levels, may enhance cholinergic signaling at both the neurotransmitter and receptor levels [43]. JH also increased mGluR5 expression, a major metabotropic glutamate receptor that regulates excitatory transmission and supports activity-dependent plasticity [44]. Because cholinergic and glutamatergic signaling interact closely during synaptic plasticity, the simultaneous increase in CHRM1 and mGluR5 may provide a molecular environment that supports activity-dependent synaptic modulation. Both CHRM1 and mGluR5 are associated with the CaMKII/ERK/CREB signaling pathways involved in synaptic plasticity [45,46]. These signaling cascades translate synaptic activity into transcriptional responses by phosphorylating CREB, thereby supporting activity-dependent gene expression underlying synaptic plasticity [47]. JH increased the phosphorylation of CaMKII, ERK, and CREB in the hippocampus. Consistent with these findings, JH also elevated the expression of the activity-dependent genes Arc and Homer1a. These immediate early genes are involved in synaptic remodeling and postsynaptic regulation, suggesting modulation of plasticity-related signaling [35]. Collectively, these findings indicate that JH may regulate signaling pathways associated with hippocampal synaptic plasticity.

Synaptic plasticity involves not only changes in intracellular signaling and gene expression but also structural remodeling at synaptic sites [48]. At presynaptic terminals, Syn1 and SYP are associated with vesicle regulation and neurotransmitter release. Syn1 facilitates vesicle mobilization, whereas SYP reflects presynaptic vesicle integrity and synapse density [49]. At postsynaptic sites, Shank3 and PSD95 function as scaffold proteins within the postsynaptic density. Shank3 contributes to dendritic spine organization, whereas PSD95 anchors glutamatergic receptors and stabilizes excitatory synapses [50]. JH increased the expression of Shank3 and Syn1 and elevated SYP and PSD95 protein levels in the hippocampus. These findings suggest that JH may support both presynaptic and postsynaptic components of hippocampal synaptic plasticity, which may contribute to the improved memory performance observed after repeated JH administration.

Because synaptic plasticity is activity-dependent, enhanced plasticity may be associated with increased neuronal activation involved in hippocampal memory processes [51]. c-FOS is widely used as a marker of neuronal activation because its expression is induced by synaptic stimulation. Increased c-FOS expression reflects activity-dependent transcriptional responses in hippocampal neurons [52]. JH administration upregulated c-FOS expression in the hippocampus. Moreover, JH increased the number of NeuN- and c-FOS-double-positive cells, indicating activation of mature neurons. These findings suggest that repeated JH administration may enhance neuronal activation in hippocampal regions involved in memory processing, consistent with the observed changes in synaptic plasticity-related markers.

The present study extends these previous findings by showing that JH improves memory-related behavioral performance, accompanied by changes in cholinergic and synaptic plasticity-related markers. Related pharmacological effects have also been reported for individual herbs included in JH and their isolated constituents. For example, Polygalae Radix and its constituent polygalasaponin XXXII have been reported to improve scopolamine-induced cognitive impairment and enhance hippocampal synaptic transmission, long-term potentiation (LTP), ERK/CREB signaling, Syn1, and brain-derived neurotrophic factor (BDNF) expression [53]. Zizyphi Semen-derived spinosin ameliorated scopolamine-induced memory impairment in passive avoidance, Y-maze, and Morris water maze tasks and increased hippocampal ERK and CREB phosphorylation [54]. Ginseng Radix-derived ginsenosides also improved scopolamine-induced memory deficits while regulating acetylcholinesterase (AChE) activity, BDNF expression, and CREB phosphorylation [55]. In addition, Angelicae Gigantis Radix-derived decursin ameliorated scopolamine-induced amnesia and inhibited hippocampal AChE activity [56]. Taken together, these previous findings provide supportive pharmacological context for the memory-related effects of JH observed in the present study.

Despite these findings, several points should be further addressed in future studies. Although JH improved memory performance in normal mice and in a scopolamine-induced memory impairment model, the present study was limited to these experimental conditions. While modified JH preparations have shown neuroprotective effects in disease-related models, the memory-enhancing effects of JH have not yet been fully characterized across diverse pathological conditions. Further studies using longer-term treatment in chronic or progressive models of cognitive dysfunction, including models of Alzheimer’s disease, are needed to determine whether JH can ameliorate persistent disease-related memory deficits beyond transient pharmacological impairment. Moreover, electrophysiological assessments, such as LTP, were not performed. Instead, synaptic strengthening was inferred from coordinated changes in molecular signaling, gene expression, and synaptic structural markers. Direct electrophysiological validation is necessary to confirm the functional effects of JH on synaptic transmission.

## 4. Materials and Methods

### 4.1. Materials

HPLC-grade acetonitrile and methanol were purchased from J.T.Baker (Radnor, PA, USA) and MS-grade formic acid from Sigma-Aldrich (St. Louis, MO, USA). The reference standards of tenuifolin, harpagoside, spinosin, liriope muscari saponin C, nodakenin, decursin and decrusinol angelate were purchased from PhytoLab (Vestenbergsgreuth, Germany). The reference standards of ginsenoside Rg1 and ginsenoside Rb1 were purchased from EDQM (Strasbourg, France). Polyvinylidene difluoride (PVDF) membrane, rabbit anti-CREB antibody (06-863), goat anti-ChAT antibody (AB144P), and mouse anti-NeuN antibody (MAB377) were purchased from Millipore (Burlington, MA, USA). Horseradish peroxidase (HRP)-conjugated mouse anti-β-actin antibody (SC-47778HRP) and rabbit anti-ERK antibody (SC-93) were purchased from Santa Cruz Biotechnology (Temecula, CA, USA). Rabbit anti-CHRM1 antibody (AMR-001) was purchased from Alomone Labs (Jerusalem, Israel). Rabbit anti-PSD-95 antibody (AB18258), rabbit anti-mGluR5 antibody (AB76316), and rabbit anti-c-FOS antibody (AB222699) were purchased from Abcam (Cambridge, UK). Rabbit anti-CaMKII antibody (#4436), rabbit anti-phospho-CaMKII antibody (#12716), rabbit anti-phospho-ERK antibody (#9101), and rabbit anti-phospho-CREB antibody (#9198) were purchased from Cell Signaling Technology (Danvers, MA, USA). Mouse anti-SYP antibody (S5768), DNZ, and all other reagents were purchased from Sigma-Aldrich (St. Louis, MO, USA), unless otherwise specified.

### 4.2. Preparation of JH

JH (batch no. L25001) was provided by Kwangdong Pharmaceutical Co., Ltd. (Gwacheon, Republic of Korea). Approximately 1 g of JH soft extract was obtained from a total of 2348.9 mg of 11 crude herbal ingredients, corresponding to an extraction yield of 42.6%. Before extraction, some of the herbal ingredients were subjected to traditional processing methods in accordance with procedures described in *Donguibogam*, a traditional Korean medical text. The crude herbal ingredients used to prepare approximately 1 g of JH soft extract were as follows: Polygalae Radix (*Polygala tenuifolia* Willd. [Polygalaceae]; processed with ginger juice; 261.1 mg), Longan Arillus (*Dimocarpus longan* Loureiro [Sapindaceae]; 261.1 mg), Rehmanniae Radix (*Rehmannia glutinosa* Liboschitz ex Steudel [Scrophulariaceae]; washed with soju [Korean diluted liquor]; 261.1 mg), Scrophulariae Radix (*Scrophularia buergeriana* Miquel [Scrophulariaceae]; 261.1 mg), Acori Graminei Rhizoma (*Acorus gramineus* Solander [Araceae]; 261.1 mg), Ginseng Radix (*Panax ginseng* C. A. Meyer [Araliaceae]; 173.9 mg), Poria Sclertum Cum Pini Radix (*Poria cocos* Wolf [Polyporaceae]; 173.9 mg), Angelicae Gigantis Radix (*Angelica gigas* Nakai [Umbelliferae]; washed with soju; 173.9 mg), Zizyphi Semen (*Zizyphus jujuba* Miller var. *spinosa* Hu ex H. F. Chou [Rhamnaceae]; stir-fried; 173.9 mg), Liriopis Tuber (*Liriope platyphylla* Wang et Tang [Liliaceae]; 173.9 mg), and Thujae Semen (*Thuja orientalis* Linné [Cupressaceae]; defatted; 173.9 mg). The resulting soft extract was stored in a tightly sealed container at 2–8 °C until further use.

### 4.3. Preparation of Standard Solutions, Preparation of Test Samples, and Standardization of JH

All reference standards were dissolved in methanol and diluted to appropriate concentrations. Samples were extracted by ultrasonic extraction for 60 min prior to analysis. For the standardization of JH, representative marker compounds were identified and quantitatively analyzed. The identification and quantification of ginsenoside Rg1 and ginsenoside Rb1 in Ginseng Radix were performed using an LC–MS/MS system. The quantitative analysis of nodakenin, decursin, and decursinol angelate in Angelicae Gigantis Radix was performed using an HPLC–UV system. The identification of tenuifolin in Polygalae Radix, harpagoside in Scrophulariae Radix, spinosin in Ziziphi Semen, and Liriope muscari saponin C in Liriopis Tuber were conducted using an LC–MS/MS system. LC–MS/MS analysis was carried out using an Agilent 1290 Infinity LC system coupled with an Agilent G6470B triple quadrupole mass spectrometer equipped with an electrospray ionization source. Chromatographic separation was achieved using an Inertsil ODS-3 column (3.0 × 100 mm, 3 μm) or a Zorbax Eclipse C18 RRHD column (2.1 × 100 mm, 1.8 μm). The mobile phase consisted of (A) water containing 0.1–0.2% formic acid and (B) acetonitrile containing 0.1–0.2% formic acid, delivered under gradient elution conditions. Analysis was performed in MRM mode, and the MRM transitions for each compound are summarized in Table 1.

HPLC–UV analysis was performed using an Agilent 1260 Infinity system equipped with a UV detector and a Phenomenex Luna C18 column (4.6 × 250 mm, 5 μm). The mobile phase consisted of (A) water and (B) acetonitrile, and separation was achieved using a gradient elution program.

### 4.4. Animals and Treatment

Six-week-old male ICR mice were obtained from Daehan Biolink Co., Ltd. (Eumseong, Republic of Korea). Animals were housed under controlled conditions (23 ± 1 °C, 60 ± 10% humidity) with a 12-h light/dark cycle. Male ICR mice were selected as an outbred strain commonly used in neurobehavioral and pharmacological research [57]. All animals had free access to food and water and were acclimated for 7 days prior to the start of the experiment. Following a 1-week acclimation period, experiments were conducted under the same housing conditions. All procedures involving animals adhered to the National Institutes of Health guidelines for laboratory animal care (2011 revision). The experimental protocol was approved by the Institutional Animal Care and Use Committee of Kyung Hee University (approval No. KHUASP(SE)-25-369 approved on 7 August 2025). Predefined humane endpoints included a body weight loss exceeding 35% associated with reduced food and water intake, or severe behavioral abnormalities interfering with normal feeding or drinking. Animals meeting these criteria were to be euthanized by cervical dislocation after anesthesia in accordance with IACUC guidelines. However, no animals met the predefined humane endpoint criteria during the study period.

To evaluate the memory-enhancing effects of JH in normal mice, animals were randomly assigned to four groups: (1) normal (*n* = 12); (2) JH 30 mg/kg (*n* = 12); (3) JH 100 mg/kg (*n* = 12); and (4) DNZ (*n* = 12, DNZ 2 mg/kg). JH (30 or 100 mg/kg) or DNZ (2 mg/kg) was administered orally via gavage using a feeding needle for 28 days. The PAT was conducted during the final two days of treatment, with training on day 27 and the retention test on day 28. Within each group, animals were further allocated into two sets (*n* = 6 per set): one set was used for immunohistochemical analyses following transcardial perfusion and fixation, whereas the other was used for molecular biological analyses, including Western blotting, using freshly collected brain tissue.

To assess the preventive effects of JH on scopolamine-induced memory impairment, mice were randomly assigned to five groups: (1) normal (*n* = 16); (2) scopolamine (*n* = 16, 1.1 mg/kg); (3) JH 30 mg/kg (*n* = 16, scopolamine 1.1 mg/kg + JH 30 mg/kg); (4) JH 100 mg/kg (*n* = 16, scopolamine 1.1 mg/kg + JH 100 mg/kg); and (5) donepezil (*n* = 13, scopolamine 1.1 mg/kg + DNZ 2 mg/kg). JH (30 or 100 mg/kg) or DNZ (2 mg/kg) was administered orally once daily for 14 consecutive days. Behavioral testing was conducted during the final 5 days of the treatment period (days 10–14), with the Y-maze performed on day 10, NORT training and testing on days 11 and 12, respectively, and PAT training and retention testing on days 13 and 14, respectively. Scopolamine (1.1 mg/kg, i.p.) was administered 30 min before the Y-maze, NORT training, and PAT training sessions. Behavioral assessments were performed using the animals assigned to each experimental group, with the number of animals included in each analysis indicated in the corresponding figure legend. Among these animals, one experimental set (*n* = 8 per group) was used for hippocampal tissue collection and subsequent Western blot analysis, whereas the remaining animals were not subjected to molecular tissue analyses.

### 4.5. Behavior Tests

#### 4.5.1. Y-Maze

The Y-maze test was conducted using a Y-shaped apparatus with three identical arms (40 cm long × 3 cm wide × 12 cm high) to evaluate working memory [58]. Each mouse was placed at the maze center and allowed to freely explore the three arms (designated A, B, and C) for 8 min. Arm entries and entry sequences were recorded. Spontaneous alternation was defined as consecutive entries into three different arms without repetition (e.g., ABC, BCA, or CBA). Arm entries and spontaneous alternations were independently recorded by two observers blinded to the experimental groups. The percentage of spontaneous alternation was calculated as follows:

$$Spontaneous\;alternation\;(\%)=\frac{Number\;of\;alternations}{Total\;number\;of\;arm\;entries-2}\times 100$$

#### 4.5.2. NORT

The NORT was conducted in a dark open-field box (45 × 45 × 45 cm) to evaluate recognition memory [59]. During the training session, each mouse was placed in the box containing two identical objects and allowed to explore freely for 4 min. After a 24 h retention interval, one familiar object was replaced with a novel object, and the mouse was again allowed to explore both objects for 4 min (test session). Exploration time for each object was recorded. Exploration was defined as directing the nose toward the object, sniffing, or touching it. Exploration times during the NORT were independently recorded by two observers blinded to the experimental groups. Recognition memory was expressed as the percentage of time spent exploring the novel object:

$$Recognition\;(\%)=\frac{Time\;spent\;on\;novel\;object}{Time\;spent\;on\;novel+familiar\;objects}\times 100$$

#### 4.5.3. PAT

The PAT was performed to evaluate fear-associated and long-term memory using a two-compartment apparatus comprising a brightly illuminated chamber (21 × 21 × 21 cm) and a dark chamber of identical size, equipped with an electrified grid floor [60]. The compartments were separated by a guillotine door. During the acquisition trial, each mouse was placed in the bright chamber, and after 10 s, the door to the dark chamber was opened. Upon full entry into the dark chamber, the door was closed, and a foot shock was delivered through the grid floor (0.6 mA for memory-enhancement assessment in normal mice and 1.4 mA for the scopolamine-induced memory impairment; duration, 3 s). After a 24 h retention interval, the retention trial was conducted under identical conditions without shock delivery. Latency to enter the dark chamber was recorded, with a maximum cutoff time of 300 s.

### 4.6. Brain Tissue Preparation

Mice were anesthetized using 2,2,2-Tribromoethanol (25 μL/g i.p., T48402) and transcardially perfused with 0.05 M phosphate-buffered saline (PBS), followed by fixation with 4% paraformaldehyde in 0.1 M phosphate buffer. Brains were carefully removed, post-fixed overnight at 4 °C, and cryoprotected in 30% sucrose in PBS until they sank. Coronal sections (25 μm thickness) were obtained using a freezing sliding microtome (Leica Microsystems Inc., Nussloch, Germany) and stored at 4 °C in a cryoprotectant solution (25% ethylene glycol, 25% glycerol, and 0.05 M phosphate buffer) until further analysis.

### 4.7. Immunohistochemistry

Brain sections were washed with PBS and treated with 1% hydrogen peroxide for 15 min to quench endogenous peroxidase activity. After washing, sections were blocked for 1 h at room temperature in a solution containing 3% normal goat or chicken serum, 2% bovine serum albumin, and 0.3% Triton X-100. Sections were then incubated overnight at 4 °C with primary antibodies against SYP, PSD-95, or ChAT. Following PBS washes, sections were incubated with appropriate biotinylated secondary antibodies (Vector Laboratories, Burlingame, CA, USA) for 1 h at room temperature. Signal amplification was performed using the VECTASTAIN Elite ABC Kit (Vector Laboratories) for 90 min, and immunoreactivity was visualized with 3,3′-diaminobenzidine (DAB). Sections were mounted on glass slides, dehydrated through graded ethanol solutions, cleared in xylene, and coverslipped using DPX mounting medium. Optical densities of SYP and PSD-95 immunoreactivity in the cornu ammonis 3 region (CA3) were quantified using image analysis software. ChAT-immunoreactive cells in the MS were counted using ImageJ (version 1.38x, NIH, Bethesda, MD, USA).

For immunofluorescence staining, sections were incubated overnight at 4 °C with primary antibodies against ChAT, c-FOS, or NeuN. After washing, sections were incubated with appropriate fluorescent secondary antibodies (Invitrogen, Carlsbad, CA, USA) for 1 h at room temperature. Nuclear staining was performed using 4′,6-diamidino-2-phenylindole (DAPI) for 20 min. Fluorescence images were acquired using a K1-Fluo confocal microscope (Nanoscope Systems, Daejeon, Republic of Korea). Quantification of ChAT/c-FOS-positive cells in the MS and NeuN/c-FOS-positive cells in the DG was performed using ImageJ software (version 1.38x).

### 4.8. Western Blotting

Mice were anesthetized with 2,2,2-tribromoethanol (25 μL/g, i.p.; T48402). After anesthesia was confirmed, the mice were euthanized by cervical dislocation. The brains were rapidly removed, and the hippocampi were dissected on ice and stored at −80 °C until further molecular analyses, including Western blotting. Protein expression was analyzed by Western blotting as previously reported [61]. Hippocampal tissues were isolated and homogenized in radioimmunoprecipitation assay (RIPA) buffer supplemented with protease and phosphatase inhibitors. Protein concentrations were determined using the Bradford assay. Equal amounts of protein (30 μg per sample) were subjected to sodium dodecyl sulfate–polyacrylamide gel electrophoresis and transferred onto PVDF membranes. Membranes were blocked for 1 h at room temperature in Tris-buffered saline containing 0.1% Tween-20 (TBST) with 5% bovine serum albumin or skim milk. Membranes were incubated overnight at 4 °C with primary antibodies, followed by incubation with HRP-conjugated secondary antibodies for 1 h at room temperature. Protein bands were visualized using an enhanced chemiluminescence (ECL) detection system and imaged using a ChemiDoc XRS+ imaging system (Bio-Rad Laboratories). Band intensities were quantified using ImageJ software (version 1.38x).

### 4.9. Quantitative Real-Time Reverse Transcription–Polymerase Chain Reaction (qRT-PCR)

Total RNA was extracted using TRIzol reagent as previously described [30]. RNA was purified using the Hybrid-R™ kit (GeneAll, Seoul, Republic of Korea), and concentration was measured using a NanoDrop ND-2000 spectrophotometer (Thermo Fisher Scientific, Waltham, MA, USA). Complementary DNA (cDNA) was synthesized from 3 μg of total RNA using TOPscript™ RT DryMIX. qRT-PCR was performed using TOPreal™ qPCR 2× PreMIX (SYBR Green; Enzynomics, Daejeon, Republic of Korea) on a CFX Connect Real-Time PCR System (Bio-Rad Laboratories, Hercules, CA, USA). Primers were synthesized by COSMO Genetech (Seoul, Republic of Korea), and the sequences are listed below; Activity-regulated cytoskeleton-associated protein (Arc): forward, 5′-CTG GGA CAG GTG ATG GAA TC-3′, reverse, 5′-TGC TGC TGC TGT TGT TGA TG-3′; Sh3 and multiple ankyrin repeat domains 3 (Shank3): forward, 5′-CTT CAG CCA GGA GGA CAA AG-3′, reverse, 5′-GCT GGT AGT TGG TGG TGA TG-3′; Synapsin 1 (Syn1): forward, 5′-AGC AGA GAG AGC CAG AGG TT-3′, reverse, 5′-TTC TGC TTG GTC AGG TTG GT-3′; Homer1a: forward, 5′-CAA CCT ATC TTC AGC ACT CG-3′, reverse, 5′-AGG AGA CTG AAG ATC TCC TC-3′; Glyceraldehyde 3-phosphate dehydrogenase (GAPDH): forward, 5′-TGA ATA CGG CTA CAG CAA CA-3′, reverse, 5′-AGG CCC CTC CTG TTA TTA TG-3′.

### 4.10. Measurement of Hippocampal Acetylcholine and Choline Levels

Mice were anesthetized, and the brains were rapidly removed. The hippocampi were dissected and collected for measurement of ACh and choline levels. Hippocampal ACh and choline concentrations were determined using a Choline/Acetylcholine Assay Kit (ab65345, Abcam, Cambridge, UK) according to the manufacturer’s instructions.

### 4.11. Statistical Analysis

Data are expressed as the mean ± standard error of the mean (SEM). Statistical analyses were conducted using GraphPad Prism 8.0 (GraphPad Software, San Diego, CA, USA). Group differences were assessed by one-way analysis of variance (ANOVA), followed by Dunnett’s multiple-comparisons test. For each ANOVA, the F statistic, numerator and denominator degrees of freedom, and corresponding exact *p*-value are provided. Individual group comparisons are reported using *p*-values adjusted by Dunnett’s test, with the reference group specified in the corresponding figure legend. Statistical significance was defined as *p* < 0.05.

## 5. Conclusions

In conclusion, the present findings demonstrate that JH improves memory performance in mice and that these effects are associated with enhanced septo-hippocampal cholinergic signaling, synaptic plasticity-related changes, and hippocampal neuronal activation. These effects were accompanied by changes in plasticity-related gene expression and synaptic markers and were further supported by the attenuation of scopolamine-induced memory impairment. Collectively, these findings provide pharmacological evidence supporting the traditional use of JH for forgetfulness and memory decline and suggest that cholinergic signaling and synaptic plasticity may contribute to its memory-enhancing effects.

## Figures and Tables

**Table 1:** MRM conditions for the six compounds.

Compound	Precusor Ion (*m*/*z*)	Product Ion (*m*/*z*)	Mode
Ginsenoside Rg1	823.5	643.5, 203.5	Positive
Ginsenoside Rb1	1131.6	789.4, 365.4	Positive
Tenuifolin	679.4	455.4, 425.3	Negative
Harpagoside	517.2	369.1, 351.1, 203.0	Positive
Spinosin	631.2	469.0, 451.0, 319.0	Positive
Liriope muscari saponin C	915.4	737.3, 575.3	Negative

## Data Availability

The raw data supporting the conclusions of this article will be made available by the authors on request.

## Supplementary Materials

The following supporting information can be downloaded at https://www.mdpi.com/article/10.3390/ph19091479/s1, Figure S1: MRM transition profiles of ginsenoside Rg1 and ginsenoside Rb1 in standard solutions and JH; Figure S2: Identification of liriope muscari saponin C in JH; Figure S3: The identification of spinosyn, harpagoside, and tenuifolin in JH.

## Abbreviations

The following abbreviations are used in this manuscript:

JH	Jangwon-hwan
ACh	Acetylcholine
HPLC	High-performance liquid chromatography
MS	Mass spectrometry
PVDF	Polyvinylidene difluoride
CREB	cAMP response element-binding protein
ChAT	Choline acetyltransferase
NeuN	Neuronal nuclei
HRP	Horseradish peroxidase
ERK	Extracellular signal-regulated kinase
CHRM1	Cholinergic receptor muscarinic 1
PSD-95	Postsynaptic density protein 95
mGluR5	Metabotropic glutamate receptor 5
c-FOS	Cellular Fos proto-oncogene
CaMKII	Calcium/calmodulin-dependent protein kinase II
SYP	Synaptophysin
DNZ	Donepezil
LC-MS/MS	Liquid chromatography–tandem mass spectrometry
ESI	Electrospray ionization
MRM	Multiple reaction monitoring
NORT	Novel object recognition test
PAT	Passive avoidance test
PBS	Phosphate-buffered saline
DAB	3,3′-Diaminobenzidine
CA3	Cornu ammonis 3 region
MS	Medial septum
DAPI	4′,6-Diamidino-2-phenylindole
DG	Dentate gyrus
RIPA	Radioimmunoprecipitation assay
TBST	Tris-buffered saline containing 0.1% Tween-20
ECL	Enhanced chemiluminescence
Arc	Activity-regulated cytoskeleton-associated protein
Shank3	SH3 and multiple ankyrin repeat domains 3
Syn1	Synapsin 1
Homer1a	Homer scaffold protein 1a
GAPDH	Glyceraldehyde-3-phosphate dehydrogenase
SEM	Standard error of the mean
ANOVA	Analysis of variance
RT	Retention time
