# Supplementary material for: Jangwon-hwan Improves Memory Performance in Mice with Enhanced Cholinergic Signaling and Synaptic Plasticity
Source: Pharmaceuticals (Basel). 2026 Sep 17;19(9):1479. doi: 10.3390/ph19091479 (PMC13610790; doi:10.3390/ph19091479)
Supplement: Supplementary file 1 [file pharmaceuticals-19-01479-s001.zip › pharmaceuticals-4564495-supplementary.pdf]

Supplementary Figure S1. MRM transition profiles of ginsenoside Rg1 and ginsenoside Rb1 in standard solutions and JH.

MRM transition profiles of ginsenoside Rg1 in the standard solution (A) and JH (B), and ginsenoside Rb1 in the standard solution (C) and JH (D). The selected precursor-to-product ion transitions used for MRM detection are indicated in each panel.

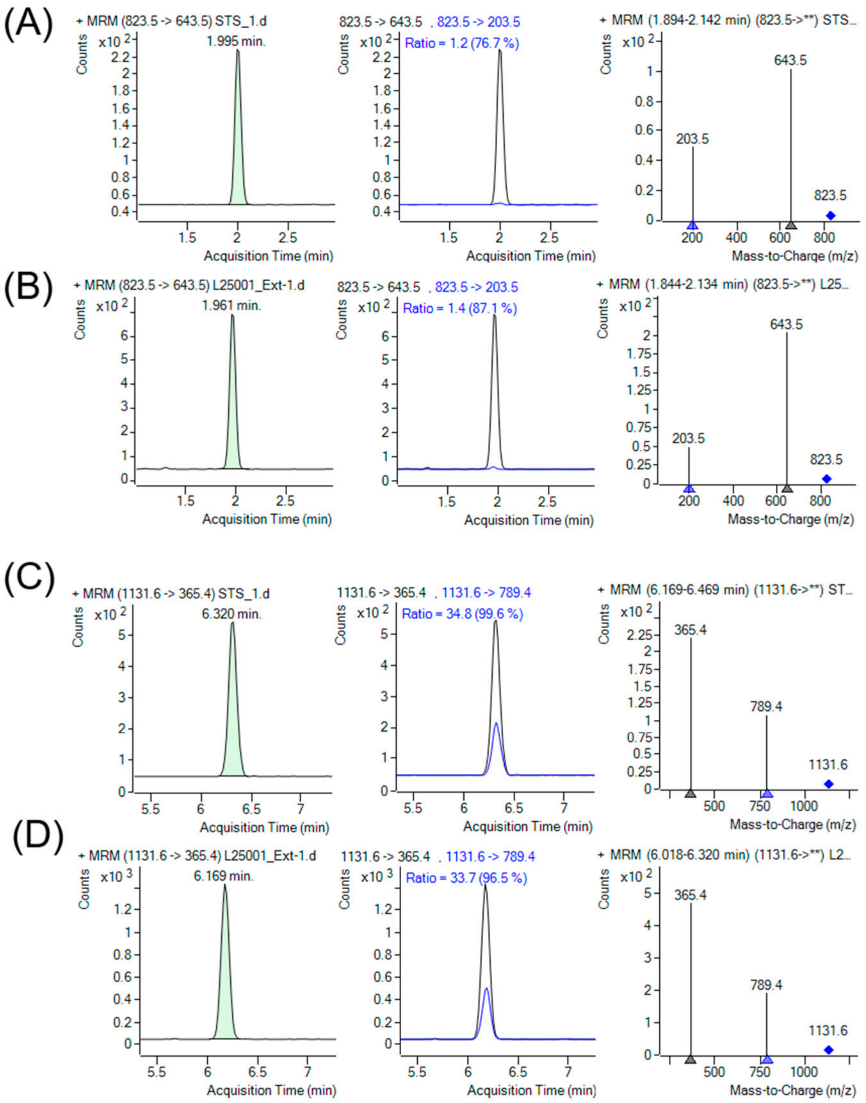

Supplementary Figure S1. MRM transitions showing precursor and product ions of ginsenoside Rg1 in the standard solution (A) and JH (B). MRM transitions showing precursor and product ions of ginsenoside Rb1 in the standard solution (C) and JH (D).

Supplementary Figure S2. Identification of liriope muscari saponin C in JH

Liriope muscari saponin C was identified in JH by LC–MS/MS analysis, showing a RT of 15.819 min consistent with that of the reference standard.

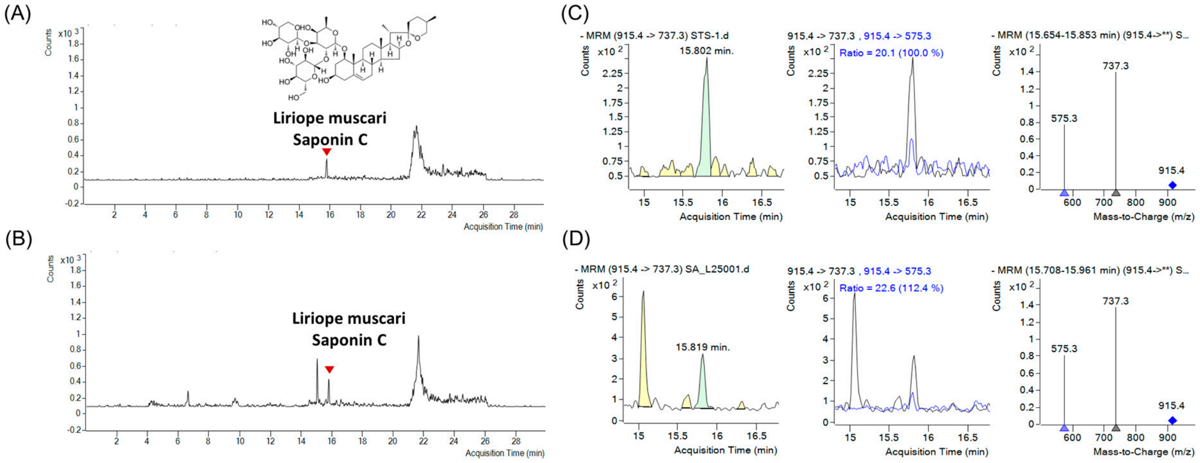

Supplementary Figure S2. LC chromatograms of Liriope muscari saponin C (RT = 15.819 min) standard solutions (A). LC–MS/MS chromatogram of JH (B). MRM transitions showing precursor and product ions of Liriope muscari Saponin C in the standard solution (C) and JH (D).

Supplementary Figure S3. The identification of spinosyn, harpagoside, and tenuifolin in JH

LC–MS/MS analysis confirmed the presence of spinosin (RT = 4.321 min), harpagoside (RT = 5.800 min), and tenuifolin (RT = 9.580 min) in JH, based on matching retention times and MS/MS fragmentation patterns with authentic reference standards.

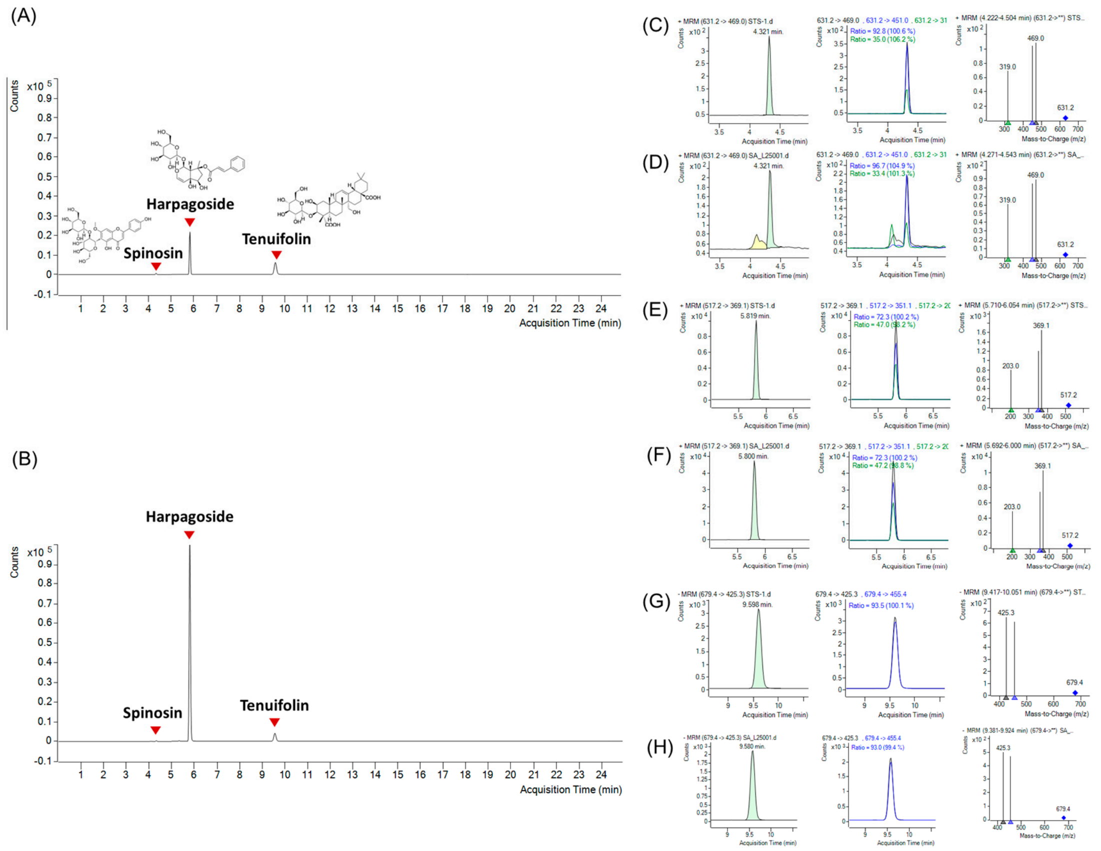

Supplementary Figure S3. LC chromatograms of spinosin (RT = 4.321 min), harpagoside (RT = 5.800 min), and tenuifolin (RT = 9.580 min) standard solutions (A). LC–MS/MS chromatogram of JH (B). MRM transitions showing precursor and product ions of spinosin in the standard solution (C) and JH (D). MRM transitions showing precursor and product ions of harpagoside in the standard solution (E) and JH (F). MRM transitions showing precursor and product ions of tenuifolin in the standard solution (G) and JH (H).
